# Psychosocial factors modify the association of frailty with adverse outcomes: a prospective study of hospitalised older people

**DOI:** 10.1186/1471-2318-14-108

**Published:** 2014-09-28

**Authors:** Elsa Dent, Emiel O Hoogendijk

**Affiliations:** Discipline of Public Health, School of Population Health, The University of Adelaide, 178 North Terrace-Terrace Towers, Adelaide, Australia; Department of General Practice & Elderly Care Medicine, EMGO + Institute for Health Care Research, VU University Medical Center, Van de Boechorststraat 7, 1081BT Amsterdam, The Netherlands

**Keywords:** Frail elderly, Psychosocial factors, Geriatric assessment/methods, Hospitalisation

## Abstract

**Background:**

Frailty increases the risk of adverse outcomes in older people. The impact of psychosocial factors on frailty and adverse clinical outcomes associated with frailty has not yet been examined in the hospital setting. The aims of this study were to: i) investigate the association between psychosocial factors and frailty, and ii) to establish whether psychosocial factors impact on the association between frailty and adverse outcomes.

**Methods:**

Data was collected from a Geriatric Evaluation and Management Unit (GEMU) in Australia. Frailty was identified using Fried’s frailty criteria. Psychosocial factors included wellbeing, sense of control (mastery), social activities, home/neighbourhood satisfaction, social relationships, anxiety and depression. Outcome measures were: mortality at 12 months, long length of GEMU stay (LOS), 1-month emergency rehospitalisation, and a higher level of care needed on discharge. Covariates adjusted for were age, gender and comorbidity.

**Results:**

The mean (SD) age of participants (n = 172) at admission was 85.2 (6.4) years, with 129 (75%) female patients. 96 (56%) patients were classified as frail, with 64 (37%) pre-frail and 12 (7%) robust. Frail patients had an increased likelihood of 12-month mortality (HR, 95% CI = 3.16, 1.36–7.33), discharge to a higher level of care (OR, 95% CI = 2.40, 1.21–4.78), long LOS (OR, 95% CI = 2.04, 1.07–3.88) and 1-month emergency rehospitalisation (OR, 95% CI = 2.53, 1.10–5.82). Psychosocial factors associated with frailty included poor wellbeing, anxiety, depression, and a low sense of control. Several psychosocial factors increased the likelihood of adverse outcomes associated with frailty, including anxiety and low ratings for: wellbeing, sense of control, social activities and home/neighbourhood satisfaction.

**Conclusions:**

Our results indicate that frail older adults with low psychosocial resources had an elevated risk of mortality, discharge to higher level care, long LOS and rehospitalisation. Consideration of psychosocial factors in comprehensive geriatric assessments will assist in patient care planning.

**Electronic supplementary material:**

The online version of this article (doi:10.1186/1471-2318-14-108) contains supplementary material, which is available to authorized users.

## Background

Amid the rapid rise in the number of older people worldwide, frailty will place an increasing pressure on health care systems. Frailty is common in older people, affecting over one quarter of older people aged 85 years or older [1]. Frailty is a multidimensional geriatric syndrome [2, 3] reflecting multi-system dysfunction [4, 5] and a reduced capacity to cope with stressors [6]. An older person inflicted with frailty has an elevated risk for multiple adverse outcomes, including mortality, nursing home admission and hospitalisation [5, 7–9] .

Frailty is well known to be linked to psychosocial factors [10, 11]. For instance, frailty was found to be related to a person’s wellbeing in a recent study of community-dwelling older people [11]. Nonetheless, despite the strong research links between frailty and psychosocial factors, very little is known about the ability of psychosocial factors to modify frailty outcomes. It could perhaps be that a frail person’s psychosocial resources act as a safeguard against adverse outcomes. Indeed, positive psychological factors, such as wellness and sense of control (mastery) have been found to shield community-dwelling older people from transitioning from a state of lower physical performance into further disability states [12].

The relationship between frailty and adverse outcomes is often studied in community-based populations [5, 13–15]. However, very little is known about this association in specific clinical settings [9] such as the hospital setting. Hospitals provide a fundamental location in which to study frailty, due to their crucial turning-point role in patient decline [16] and their high prevalence of frailty [8, 16]. No study has yet investigated the modifying effect psychosocial factors have on frailty outcomes in hospitals.

The aims of this hospital-based study were: a) to investigate the association between psychosocial factors and frailty, and b) to establish whether psychosocial factors impact on the association between frailty and adverse outcomes. Study outcomes included mortality, admission to higher level care, long length of stay (LOS) and 1-month emergency rehospitalisation.

## Methods

### Setting and participants

In this prospective, observational study, consecutive patients aged 70 years and older were recruited between October 22, 2010 to December 23, 2011 from the 20-bed sub-acute care Geriatric Evaluation and Management Unit (GEMU) at The Queen Elizabeth Hospital (TQEH), South Australia. TQEH is a public hospital in the western suburbs of Adelaide, with a coverage base of 250,000 people. All eligible patients or their authorized proxy were approached to obtain written informed consent. Patient exclusion criteria were language barrier without a proxy, physician advised against inclusion (elder-abuse, physically aggressive, medically unwell and/or infectious) and dementia/unresolved delirium without a proxy. Full methodology details have been described elsewhere [17]. Ethical guidelines from the Australian Code for the Responsible Conduct of Research were adhered to. TQEH Ethics Committee approved the study protocol (Protocol Number: 2010105).

### Measures

Baseline data were collected during the first 72 hours of a patient’s admission to the GEMU in the following order: (i) patient (or proxy) interview, (ii) measurement of frailty components and (iii) review of patient medical charts. Patient (or proxy) interview was used to collect data on socio-demographic variables (birthplace, marital status, household structure and carer use) and psychosocial factors. Patient medical charts were reviewed to obtain data on cognitive ability assessed by the Mini-Mental State Examination (MMSE) [18] and co-morbidity (Charlson’s Comorbidity Index (CCI) [19]).

Frailty was measured at baseline by the first author, using Fried’s frailty criteria for identification [5]. Fried’s criteria classifies frailty as three or more of weakness (low grip strength), slowness (slow walking speed), weight loss (unintentional), low physical activity and self-reported exhaustion [5]. Both exhaustion and weight loss (>4.5 kg over the last year), were defined using Fried’s original cut-off scores [5]. Low grip strength (<18 kg women and < 30 kg men), and low physical activity were defined using the criteria from the Australian-based Frailty Intervention Trail [20]. Slow walking speed was defined as unable to walk 6 m in 30s, with or without a walking aid [21]. Scoring for each frailty component is described in the Additional file 1.

### Psychosocial measures

Psychosocial measures were also performed at admission. Anxiety was measured using the Geriatric Anxiety Inventory Short Form (GAI-SF) [22] and depressive symptoms using the Geriatric Depression Scale-Short Form (GDS-Short Form) [23]. Five categories of the Older People’s Quality of Life questionnaire were used to form the psychosocial variables in our study: (i) wellbeing; (ii) sense of control (mastery); (iii) social activities; (iv) enjoyment of home and neighbourhood and (v) social relationships [24]. Each of these five categories contained 4–5 questions, with patients indicating the extent of their agreement/disagreement with each question using a Likert scale response: strongly agree, agree, neither agree or disagree, disagree or strongly disagree [24]. The response for each question was then given a score from 1–5, then scores for each category were computed. The lowest quartile for each psychosocial variable was considered as a poor response.

### Outcomes

Four outcome measures were studied:

(i) Mortality at 12 months post-discharge, determined using Australian Death Registry data and the electronic Open Architecture Clinical Information System (OACIS).
(ii) A higher level of care needed on hospital discharge. Higher level of care was defined as a move to a location other than home on hospital discharge, and included residential care admission, a move within residential care from low to high level care, sub-acute care (multidisciplinary care in which the main goal is maintaining patient QOL and function) and admission to a transition care program (a short-term, government-funded therapy and support program for older patients discharged from hospital, which aims to avoid early admission to residential care). Nine patients were excluded from this analysis: two because they were already in high level care before hospital admission and 7 because they died before hospital discharge.
(iii) Long GEMU length of stay (LOS), defined as > 12 days, the median LOS of GEMU patients.
(iv) Emergency rehospitalisation at 1 month post-hospitalisation, defined as in-patient admission to TQEH emergency department as determined using OACIS. The 1-month time period was chosen according to previous research, as most adverse events occur during this time-frame [9].

### Statistics

All continuous variables considered in this study were normally distributed. Due to the limited number of people classified as robust, a ‘not frail’ category, combining the pre-frail and robust groups was formed. To analyse the association of gender and age on frailty, Chi-square and t-tests were performed, respectively. The association of psychosocial variables with frailty was determined using a binary logistic regression model for each variable, with each model controlling for age, gender and CCI.

To determine factors affecting mortality, two Cox Regression models were performed for each variable: Model 1 adjusted for age, gender and CCI; Model 2 adjusted for age, gender, CCI and frailty status. Education level was not adjusted for due to the homogeneity of the low education level of patients. To determine factors associated with admission to higher level care, long LOS and 1-month emergency rehospitalisation, two binary logistic regression analyses were performed for each variable, with these models containing the same variables as the Cox Regression analyses. To investigate the impact of low levels of each psychosocial variable on study outcomes in those patients classified as frail, interaction effects of frailty with each psychosocial variable were analysed. All regression models were checked for collinearity using Variance Inflation Factors. All data was analysed using SPSS 21.0 (SPSS Inc, Chicago, Illinois, USA) with statistical significance set at P < 0.05.

## Results

427 new patients were admitted to the GEMU during the study timeframe, of which 172 patients were recruited. Exclusion reasons were: unresolved delirium or dementia (n = 77), language barrier (n = 67), infectious (n = 11), missed by researcher (n = 4), did not wish to participate (n = 63) and the treating physician advised against study inclusion (for elder abuse, physical aggression or medically unwell) (n = 33) [17]. The mean (SD) age of participants at admission was 85.2 (6.4) years, with 129 (75%) female patients. Over half of all patients (56%) were classified as frail on hospital admission, with 64 (37%) pre-frail and 12 (7%) robust. The median length of hospitalisation before GEMU entry was 4 days. 74 (57%) of female patients and 22 (51%) of male patients were classified as frail on GEMU entry, although this gender difference in frailty prevalence was not statistically significant (P = 0.069). There was no statistical difference between the age (SD) of frail [85.6 (6.1) years] and non-frail patients [84.6 (6.8) years] (P = 0.234). Only 15 (9%) of all patients completed high school.

Table 1 shows demographic and psychosocial variables of patients on GEMU admission and their association with frailty, as determined by binary regression analyses controlling for age, gender and co-morbidity. From this table, it can be observed that frail patients were more likely to have high anxiety, depression, poor wellbeing, a low control over life and to require assistance from a carer.

**Table 1:** **Demographic and psychosocial characteristics of patients on admission, and their association with frailty (n = 172)** ^**†**^

Characteristic	Overall ***n***(%)	Frailty (n = 96)
		(OR, 95% CI, P)
***Demographic characteristics***		
Caregiver-informal or paid	124 (72)	**3.22 (1.58–6.57), 0.001**
Birthplace (Australia)	118 (69)	1.02 (0.32–2.01), 0.994
Lives alone	105 (61)	1.47 (0.77–2.84), 0.244
Private health insurance	62 (36)	1.43 (0.75–2.72), 0.276
Married or defacto	59 (34)	1.03 (0.52–2.03), 0.943
***Psychosocial Characteristics***		
High anxiety severity (GAI-SF score ≥ 3/5)	66 (38)	**2.29 (1.17–4.48), 0.015**
Depression (GDS-SF Score > 6)	52 (30)	**2.66 (1.29–5.47), 0.008**
Wellbeing (lowest quartile)	n.a.	**4.36 (1.81–10.54), 0.001**
Sense of control (lowest quartile)	n.a.	**3.88 (1.73–8.70), 0.001**
Leisure and social activities (lowest quartile)	n.a.	2.24 (0.77–6.52), 0.141
Enjoyment of home/neighbourhood (lowest quartile)	n.a.	1.45 (0.72–2.91), 0.296
Social relationships (lowest quartile)	n.a.	1.34 (0.63–2.84), 0.444

^**†**^Each variable was computed as a separate binary logistic regression model, controlling for age, gender and Charlson’s Comorbidity Index. Outcomes significantly associated with frailty are highlighted in bold text. n = 172.

n.a. = not applicable.

Results of the logistic regression analyses showing the association of frailty with study outcomes is displayed in Table 2. Evident from this table is that frail patients had an increased likelihood of 12-month mortality (HR, 95% CI = 3.16, 1.36–7.33), discharge to a higher level of care (OR, 95% CI = 2.40, 1.21–4.78), long LOS (OR, 95% CI = 2.04, 1.07–3.88) and 1-month emergency rehospitalisation (OR, 95% CI = 2.53, 1.10–5.82) after adjustment for age, gender and co-morbidity (Model 1). Also presented in Table 2 are the associations of psychosocial factors with adverse outcomes. After adjustment for age, gender and co-morbidity (Model 1), psychosocial factors found to be associated with 12-month mortality included a poor sense of control (HR, 95% CI = 2.97, 1.29–6.83); associated with discharge to higher level care included poor wellbeing (OR, 95% CI = 2.81, 1.30–6.06), poor sense of control, (OR, 95% CI = 3.22, 1.54–6.72) and a low level of social activities (OR, 95% CI = 3.36, 1.01–11.22). When frailty was added as a covariate (Model 2), psychosocial factors were no longer predictive of mortality, with the exception of both poor wellbeing and poor sense of control which remained associated with discharge to higher level care. No psychosocial factors were associated with long LOS or 1-month rehospitalisation for either Model 1 or 2.

**Table 2:** **The association of frailty and psychosocial factors with outcomes**

	12-Month mortality ( ***n***=40 of 172)		Discharged to higher level care ( ***n***=70 of 163)		Long GEMU LOS (> 12 days) (n=97 of 172)		1-month emergency rehospitalisation (n=38 of 163)	
	Model 1	Model 2	Model 1	Model 2	Model 1	Model 2	Model 1	Model 2
	HR (95% CI)	HR (95% CI)	OR (95% CI)	OR (95% CI)	OR (95% CI)	OR (95% CI)	OR (95% CI)	OR (95% CI)
Frailty	**3.16 (1.36-7.33)**	n.a.	**2.40 (1.21-4.78)**	n.a.	**2.04 (1.07-3.88)**	n.a.	**2.53 (1.10-5.82)**	n.a.
***Demographic characteristic***								
Lives alone	0.79 (0.39-1.60)	0.83 (0.42-1.68)	1.01 (0.52-1.97)	1.12 (0.56-2.22)	1.01 (0.53-1.91)	1.08 (0.56-2.06)	1.10 (0.51-2.39)	1.02 (0.46-2.23)
unmarried	1.14 (0.51 -2.52)	0.91 (0.38-2.17)	0.92 (0.46-1.86)	0.90 (0.44-1.85)	1.51 (0.77-2.97)	1.52 (0.77-3.03)	1.89 (0.85-4.20)	1.90 (0.84-4.29)
***Psychosocial characteristic*** ^**†**^								
Anxiety (GAI-SF>3)	1.79 (0.85-3.78)	1.56 (0.74-3.26)	1.25 (0.64-2.46)	1.59 (0.78-3.26)	0.72 (0.38-1.36)	0.87 (0.44-1.69)	0.70 (0.32-1.53)	0.83 (0.37-1.87)
Depression (GDS-SF>6)	1.50 (0.65-3.45)	1.49 (0.66-3.37)	1.07 (0.53-2.14)	0.88 (0.43-1.81)	1.39 (0.72-2.70)	1.21 (0.61-2.40)	1.26 (0.57-2.80)	1.02 (0.44-2.33)
Wellbeing (poor)	1.91 (0.88-4.15)	1.84 (0.86-3.92)	**2.81 (1.30-6.06)**	**2.26 (1.01-5.04)**	1.28 (0.52-3.15)	0.96 (0.37-2.45)	2.12 (1.00-4.50)	1.70 (0.77-3.73)
Sense of control (poor)	**2.97 (1.29-6.83)**	2.30 (0.94-5.64)	**3.22 (1.54-6.72)**	**2.66 (1.25-6.00)**	2.17 (1.07-4.41)	1.80 (0.86-3.76)	2.02 (0.88-4.64)	1.68 (0.71-3.94)
Social activities (poor)	0.92 (0.20-4.29)	0.77 (0.17-3.51)	**3.36 (1.01-11.22)**	2.94 (0.86-10.05)	1.04 (0.36-3.03)	0.88 (0.29-2.63)	0.40 (0.12-1.33)	0.31 (0.85-1.09)
Social relationships (poor)	1.99 (0.79–5.04)	1.68 (0.66-4.31)	1.87 (0.86-4.09)	2.01 (0.91-4.46)	0.64 (0.30-1.37)	0.60 (0.28-1.30)	0.68 (0.26-1.78)	0.63 (0.24-1.67)

Model 1 controlled for age, gender and Charlson’s Comorbidity Index; Model 2 controlled for age, gender Charlson’s Comorbidity Index and Frailty Status measured using Fried’s criteria.

^†^For each psychosocial factor with a ‘poor’ ranking, poor was considered as the lowest quartile.

*Abbreviations*: *HR* Hazard Ratio, *CI* confidence interval, *n.a.* not applicable, *GAI-SF* Geriatric Anxiety Inventory – Short Form, *GDS-SF* Geriatric Depression Scale – Short Form, *LOS* Length of Stay, *GEMU* Geriatric Evaluation and Management Unit. Significant factors are highlighted in **BOLD**.

Table 3 presents the interaction effects of frailty with psychosocial factors. From this table it can be seen that frail people with poor psychosocial resources were more likely to encounter adverse outcomes than frail people with good psychosocial resources. For instance, frail people with poor sense of control had an increased likelihood of all four study outcomes compared with their frail peers that had a good sense of control: 12-month mortality (HR, 95% CI = 3.92, 1.67–9.24), discharge to higher level care (OR, 95% CI = 3.29, 1.46–7.39), long LOS (OR, 95% CI = 2.34, 1.08–5.09) and 1-month emergency rehospitalisation (OR, 95% CI = 2.46, 1.02–5.98). Frail people with poor wellbeing had an increased likelihood of mortality, discharge to higher level of care and long LOS than frail people with good wellbeing. Similarly, frail people reporting low enjoyment of their home and neighbourhood were at an higher likelihood for mortality, discharge to higher level care and 1-month emergency rehospitalisation. Additionally, those frail people with low levels of social activities had higher odds of both 12-month mortality and discharge to higher level care. Lastly, frail people who were unmarried were more likely to have a longer LOS than those who were married, and frail people with anxiety were at a higher risk for 12-month mortality.

**Table 3:** **The interaction effects of frailty with psychosocial and demographic factors**

	12-Month mortality ( ***n***=40 of 172) HR (95% CI)	Discharge to higher level care ( ***n***=70 of 163) OR (95% CI)	Long GEMU LOS (> 12 days) (n=97 of 172) OR (95% CI)	1-month emergency rehospitalisation (n=38 of 163) OR (95% CI)
***Demographic characteristics***				
Frailty × Living Alone	1.51 (0.70-3.24)	1.55 (0.80-2.99)	1.15 (0.59-2.21)	1.94 (0.88-4.26)
Frailty × Unmarried	2.03 (0.92-4.48)	1.56 (0.72-3.54)	**2.88 (1.26-6.58)**	1.97 (0.80–4.87)
**Psychosocial characteristics** ^**§**^				
Frailty × Anxiety (GAI-SF Score > 3)	**2.69 (1.21-5.98)**	1.57 (0.77-3.20)	1.57 (0.77-3.17)	1.56 (0.68-3.61)
Frailty × Depression (GDS-SF Score > 6)	1.32 (0.50-3.48)	1.44 (0.68-3.05)	1.28 (0.61-2.67)	1.66 (0.71-3.91)
Frailty × Poor Wellbeing	**4.70 (1.85-11.96)**	**2.63 (1.15-6.01)**	**2.68 (1.17-6.13)**	1.53 (0.60-3.93)
Frailty × Low Sense of Control	**3.92 (1.67-9.24)**	**3.29 (1.46-7.39)**	**2.34 (1.08-5.09)**	**2.46 (1.02-5.98)**
Frailty × Low Social Activities	**2.73 (1.21-6.17)**	**2.82 (1.48-5.38)**	1.87 (0.99-3.54)	1.69 (0.76-3.76)
Frailty × Poor Home/Neighbourhood	**2.94 (1.19-7.25)**	**2.36 (1.03-5.41)**	1.51 (0.73-3.11)	**2.47 (1.09-5.64)**
Frailty × Poor Social Relationships	0.71 (0.18-2.75)	1.13 (0.46-2.76)	1.28 (0.53-3.07)	1.28 (0.53-3.07)

Each regression model controlled for age, gender, co-morbidity and the main effects of frailty and demographic/psychosocial factors.

^§^For each psychosocial factor with a ‘poor’ ranking, poor was considered as the lowest quartile.

*Abbreviations*: *HR* Hazard Ratio, *CI* confidence interval, *GAI-SF* Geriatric Anxiety Inventory – Short Form, *GDS-SF* Geriatric Depression Scale – Short Form, *LOS* Length of Stay, *GEMU* Geriatric Evaluation and Management Unit. Significant factors are highlighted in **BOLD**.

## Discussion

In this study of older people hospitalised in a GEMU, frailty was associated with 12-month mortality, admission to higher level care, long LOS and 1-month emergency rehospitalisation. Psychosocial variables found to be associated with frailty included anxiety, poor wellbeing, depression and low sense of control (mastery). We also determined whether psychosocial factors modified outcomes of frailty. We found that frail older people with poor psychosocial resources had a significant increase in the probability of adverse outcomes. Specifically, frail people had increased odds of both 12-month mortality and discharge to higher level of care if they had a low levels of: wellbeing, sense of control, social activities and enjoyment of their home/neighbourhood. If they had high anxiety symptoms, they were also more likely to die in the 12-months post-hospitalisation. Frail patients were also at increased risk of a long LOS if they had low levels of wellbeing and sense of control. Furthermore, frail patients with poor levels for sense of control and enjoyment of their home/neighbourhood were more likely to re-admitted to the emergency department 1-month post-hospitalisation than frail patients with good levels for these psychosocial variables.

To our knowledge, this is the first paper to investigate the association of frailty with psychosocial resources in the hospital setting. Our finding that psychosocial factors were associated with frailty supports findings from community based studies. For instance, frailty has been found to associate with anxiety, depression and mastery in community-dwelling older people [10, 11, 25]. Frailty in our study, however, was not found to be associated with social activities, social relationships or enjoyment of home/neighbourhood. This finding is in agreement with a recent study of Mexican community-dwelling older people in which a low quality of social networks, such as having no friends or relatives living in the same neighbourhood, was not associated with frailty [26]. However, other studies have found a relationship between these societal factors and frailty [27], suggesting a population effect may be present.

In the present study, frail individuals were over three times as likely to need a carer as their non-frail counterparts. This high reliance on a carer emphasises the importance of actively involving informal caregivers (such as a patient’s family) in care decision-making practices. Informal caregivers have previously been found to play an essential, yet often overlooked, role in ensuring favourable post-hospital outcomes in frail older people [28].

Also in this study, frail patients had a higher likelihood of all study outcomes. This ability of frailty to predict adverse outcomes in hospitalised older people is supported by recent studies, which have found frailty to be predictive of mortality [8] admission to higher level care [9], long LOS [8, 29], and rehospitalisation [9]. Being able to predict adverse outcomes is important clinically to guide patient care, including planning for surgical and medical treatment [30]. As such, frailty assessment, along with an evaluation of a patient’s psychosocial resources is highly recommended as part of a patient’s Comprehensive Geriatric Assessment (CGA).

This paper is also the first, to our knowledge, to investigate the effect psychosocial resources have on frailty outcomes in hospitalised older people. We found that several psychosocial factors were found to modify frailty outcomes. For instance, sense of control was associated with all four study outcomes as an effect modifier of frailty. This finding supports recent findings from the Longitudinal Aging Study Amsterdam, in which sense of control was found to shield people with low physical performance against nursing home admission [12]. This shielding effect could be due to the adaptive ability of a frail person to cope with their physical deterioration, perhaps as a result of their prior experiences with decline [12, 31]. Enjoyment of home/neighbourhood was also found to be an effect modifier of frailty for all outcomes in our study, with the exception of LOS. It could perhaps be that a person’s perception of their neighbourhood buffers against adverse health effects, given that positive neighbourhood perception has been strongly linked with health and functioning in community-dwelling older people [32]. Wellbeing was also found to be an effect modifier of all frailty outcomes in our study, with the exception of rehospitalisation. Our finding is in line with results from the Canadian Study of Health and Ageing in which poor wellbeing was linked with mortality [11].

Frailty prevalence was high in our study (56%). This prevalence is much higher than that reported in two recent hospital-based studies using Fried’s criteria (33% and 23%) [8, 33]. Our high frailty prevalence is likely due to the high average age of patients included in our study. Of note, actual frailty prevalence in our study may in fact be lower than upon hospital admission, given that the median number of days in acute care before GEMU (sub-acute care) entry was 4 days. It is also likely that patients recovering from acute illness/injury and/or those with co-morbidity would be more likely to be classified positive for Fried’s criteria components, including ‘exhaustion’ and ‘slow walking speed’.

In the present study, frailty prevalence was not significantly associated with age or gender, which is in contrast to most frailty studies [5, 10]. This lack of association could likely reflect the rehabilitation nature of the GEMU and the pre-selection of GEMU patients from acute care. Indeed, a recent study of geriatric rehabilitation patients also found no age or gender difference with regards to frailty [29].

Using frailty measurements to predict patient outcomes has gained popularity in very recent times [8, 17, 29, 33–41], including the use of Fried’s frailty criteria as a predictor of patient outcomes in both the acute care setting [33] and in medical wards [8, 41]. Being able to predict adverse clinical outcomes is of crucial importance for a frailty definition [42], and has been deemed to be the most important area to assist in establishing an international standard definition for frailty [43]. Moreover, the ability to predict patient outcomes is important for patient care planning [8, 44]. It must be noted that identifying frail patients should not used to deny older people treatment; rather it should be used to optimise patient treatment and prevent unnecessary harm [45].

Strengths of the present study include the comprehensive dataset and the inclusion of patients with dementia. Study limitations include the small sample size and the potential information bias due to patient’s families answering questions for patients with dementia and/or language barriers. No inference of causation can be made in the study. For example, it could equally be that frailty led to psychosocial decline or vice versa [11]. Furthermore, this study did not account for any changes in psychosocial resources post-hospitalisation, which could have influenced outcome results. Study results also lack generalisation to other populations of older people, as only one study location was used.

Results from this study highlight the fundamental role that psychosocial factors play in modifying outcomes of frail older people. Importantly, psychosocial factors, rather than being rigid and resistant to change, can be amenable to intervention. For example, a recent study of hospitalised older patients found that extending social support in hospitals as well as transitional support from hospital to home post-discharge, resulted in improvements in patient QOL, social functioning and vitality [46]. Future research should focus on increasing psychosocial resources in hospitalised older people. One way to increase access to these resources is to assign case-managers to each patient, whose role involves organising support services for patients both during and after hospitalisation: an approach which has recently been found to be cost-effective [46]. There is also a need for future studies to consider three time-points at follow-up in order to assess which comes first: frailty or psychosocial decline. Furthermore, in studies of frail older people, psychosocial outcomes in addition to clinical outcomes should be considered, given that psychosocial needs are often not met in this population group [47].

## Conclusion

Our results indicate that frail older adults with low psychosocial resources had an elevated risk of mortality, discharge to higher level care, long LOS and rehospitalisation. Consideration of psychosocial factors in comprehensive geriatric assessments will assist in patient care planning.

## Electronic supplementary material

Additional file 1: **Fried’s Frailty Criteria used for the study.** (DOC 34 KB)
