# Supplementary material for: Psychosocial factors modify the association of frailty with adverse outcomes: a prospective study of hospitalised older people
Source: BMC Geriatr. 2014 Sep 28;14:108. doi: 10.1186/1471-2318-14-108 (PMC4190287; doi:10.1186/1471-2318-14-108)
Supplement: Supplementary file 1 — Additional file 1: Fried’s Frailty Criteria used for the study. (DOC 34 KB) [file 12877_2014_1043_MOESM1_ESM.doc]

**Additional file 1**

Fried’s Frailty Criteria used for the study

|  | **Component** | **Details** |
| --- | --- | --- |
| **1** | **Unintentional Weight Loss**  (as per Fried’s original scale) | Have you lost more than 10 pounds (4.5 kg) unintentionally in the past year? (ie not due to dieting or exercising)?  *An answer of “yes” is a positive score for the Weight Loss Component* |
| **2** | **Exhaustion**    (as per Fried’s original scale) | *Read to the patient* *the statement:* “I felt that everything I did was an effort”. *Ask the patient:* “how often in the last week did you feel this way”? Circle response.   1. = Rarely or none of the time 2. = Some or Most of the time (1-2 days) 3. = A moderate amount of the time (3-4 days) 4. = Most of the time   Then read to the patient the statement “I could not get going”. *Ask the patient:* “how often in the last week did you feel this way”? Circle response.   1. = Rarely or none of the time 2. = Some or Most of the time (1-2 days) 3. = A moderate amount of the time (3-4 days) 4. = Most of the time   *A positive score for the Exhaustion Component = Score of 2 or 3 to any of the above questions.* |
| **3** | **Low Physical Activity**  (as per the Frailty Intervention Trial’s^†^ definition for low physical activity) | In the past 3 months:   - Did not perform weight bearing physical activity - Spent more than 4 hours per day sitting - Went for a short walk once per month or less   *If all 3 boxes are ticked, then this is a positive score for the Low Physical Activity component.* |
| **4** | **Slow walking speed**  (as per the Elderly Mobility Scale) | The inability to walk 6m under 30s, with or without a walking aid, was classified as a slow walking speed, as per the Elderly Mobility Scale, which was used routinely in GEMU patients. |
| **5** | **Weakness**  (as per Fried’s criteria, with cut-off scores for grip strength defined as as per the Frailty Intervention Trial) | Grip strength was measured using a Grip Strength Dyanometer, with the best of 3 attempts recorded. A postitive score for    *Weakness was defined as a grip strength <30kg for males and <18kg for females*. |

^†^ The Frailty Intervention Trial (FIT), as per the present study, was an Australian-based study

Abbreviations: GEMU = Geriatric Evaluation and Management Unit
